# Small Myristoylated Protein-3, Identified as a Potential Virulence Factor in *Leishmania amazonensis*, Proves to be a Protective Antigen against Visceral Leishmaniasis

**DOI:** 10.3390/ijms19010129

**Published:** 2018-01-03

**Authors:** Marcelo P. Oliveira, Vívian T. Martins, Thaís T. O. Santos, Daniela P. Lage, Fernanda F. Ramos, Beatriz C. S. Salles, Lourena E. Costa, Daniel S. Dias, Patrícia A. F. Ribeiro, Mônica S. Schneider, Ricardo A. Machado-de-Ávila, Antônio L. Teixeira, Eduardo A. F. Coelho, Miguel A. Chávez-Fumagalli

**Affiliations:** 1 Programa de Pós-Graduação em Ciências da Saúde: Infectologia e Medicina Tropical, Faculdade de Medicina, Universidade Federal de Minas Gerais, Belo Horizonte 30130-100, Minas Gerais, Brazil; marcelloperdigao@outlook.com (M.P.O.); viviantamietti@yahoo.com.br (V.T.M.); thaisteoli@gmail.com (T.T.O.S.); danipagliara@hotmail.com (D.P.L.); fe.fonsecaramos@gmail.com (F.F.R.); sallesbcs@gmail.com (B.C.S.S.); lourena.costa@yahoo.com.br (L.E.C.); daniel-sdias@hotmail.com (D.S.D.); patty-fernandes@hotmail.com (P.A.F.R.); monicasschneider@yahoo.com.br (M.S.S.); altexr@gmail.com (A.L.T.); miguel.fumagalli@pq.cnpq.br (M.A.C.-F.); 2 Programa de Pós-Graduação em Ciências da Saúde, Universidade do Extremo Sul Catarinense, Criciúma 88806-000, Santa Catarina, Brazil; r_andrez@yahoo.com.br; 3 Laboratório Interdisciplinar de Investigação Médica, Faculdade de Medicina, Universidade Federal de Minas Gerais, Belo Horizonte 30130-100, Minas Gerais, Brazil; 4 Neuropsychiatry Program, Department of Psychiatry and Behavioral Sciences, McGovern Medical School, The University of Texas Health Science Center at Houston, 1941 East Road, Houston, TX 77041, USA; 5 Departamento de Patologia Clínica, do Colégio Técnico (COLTEC), Universidade Federal de Minas Gerais, Belo Horizonte 31270-901, Minas Gerais, Brazil

**Keywords:** visceral leishmaniasis, bioinformatics, small myristoylated protein-3, peripheral blood mononuclear cells, immune response, vaccine

## Abstract

In a proteomics approach conducted with *Leishmania amazonensis*, parasite proteins showed either an increase or a decrease in their expression content during extensive in vitro cultivation, and were related to the survival and the infectivity of the parasites, respectively. In the current study, a computational screening was performed to predict virulence factors among these molecules. Three proteins were selected, one of which presented no homology to human proteins. This candidate, namely small myristoylated protein-3 (SMP-3), was cloned, and its recombinant version (rSMP-3) was used to stimulate peripheral blood mononuclear cells (PBMCs) from healthy subjects living in an endemic area of leishmaniasis and from visceral leishmaniasis patients. Results showed high interferon-γ (IFN-γ) production and low levels of interleukin 10 (IL-10) in the cell supernatants. An in vivo experiment was then conducted on BALB/c mice, which were immunized with rSMP-3/saponin and later challenged with *Leishmania infantum* promastigotes. The rSMP-3/saponin combination induced high production of protein-specific IFN-γ, IL-12, and granulocyte-macrophage colony-stimulating factor (GM-CSF) by the spleen cells of the immunized mice. This pattern was associated with protection, which was characterized by a significant reduction in the parasite load in distinct organs of the animals. Altogether, these results have revealed that this new virulence factor is immunogenic in both mice and humans, and have proven its protective efficacy against visceral leishmaniasis in a murine model.

## 1. Introduction

Leishmaniasis is a vector-borne disease complex caused by protozoan parasites of the genus *Leishmania*, which presents a high morbidity and mortality worldwide. Around 380 million people are at risk of infection in 98 countries, with approximately 1.5–2.0 million cases being registered annually [1]. The outcome of infection depends mainly of the immune response of the hosts and the virulence of the parasite strain [2]. In addition, the disease has gained relevance in human immunodeficiency virus (HIV)-infected patients as an opportunistic infection in areas where both pathogens are endemic [3].

Currently, about 54 *Leishmania* species are known, and at least 21 of these can cause human disease [4]. Some parasite species cause a chronic and slow-to-heal disease called tegumentary leishmaniasis (TL), while others spread to distinct organs, such as the spleen, liver, and bone marrow, in turn causing visceral leishmaniasis (VL). In Brazil, leishmaniasis is caused by at least six different *Leishmania* species. Among these, *Leishmania amazonensis* is of particular interest, since it is capable of causing both TL and VL [5,6]. By contrast, *L. infantum* is the main parasite species responsible for the cases of VL [7].

Current control methods for leishmaniasis are mainly focused on the diagnosis and treatment of disease in humans. There is no gold standard test to diagnosis human leishmaniasis, and a combination of clinical assessment and parasitological tests is needed [8]. Disease treatment also presents challenges due to high costs and/or toxicity of drugs, in addition to parasite resistance [9]. In this context, the development of prophylactic vaccination to protect against VL has gained importance [10]. Nevertheless, the development of a vaccine to prevent against disease has been hampered by different factors, such as the need to associate immune adjuvants, immunization schedules, variable efficacy of the employed antigens, among others [11].

The immune response associated with the protection against *Leishmania* infection is dependent on the development of a Th1-type immunity marked by the production of cytokines, such as interferon-γ (IFN-γ), tumor necrosis factor-α (TNF-α), interleukin 12 (IL-12), and granulocyte-macrophage colony-stimulating factor (GM-CSF), which activate macrophages to produce nitric oxide (NO). Conversely, susceptibility has been associated with the production of cytokines, such as IL-4, IL-10, IL-13, and transforming growth factor-β (TGF-β), which inhibit the Type 1 T helper cell (Th1) response, thus allowing the development of the disease [12,13]. Peripheral blood mononuclear cells (PBMCs) from VL patients developing the active disease exhibit a profile marked by the suppression of the Th1 response, coupled with the production of high levels of IL-10. By contrast, PBMCs from asymptomatic patients or healthy people living in endemic areas of VL usually produce high levels of IFN-γ after antigenic stimuli [14,15].

Biotechnological approaches, such as proteomics, allow for the identification of proteins expressed in distinct microorganisms and pathogens, which can be evaluated as diagnostic markers, vaccine candidates, and/or drug targets against diseases [16]. Recently, a proteomic study involving *L. amazonensis* showed that an in vitro cultivation of stationary promastigotes for 150 days induced alterations in the protein content of the parasites, and a reduction in their infectivity was observed when both in vitro and in vivo experiments were performed [17]. Some of these molecules decreased in expression, while others showed an increase, making it possible to infer their role in the in vivo infectivity and the in vitro metabolism of the parasites, respectively.

The purpose of the present study was to perform a computational screening of these proteins that increased or decreased their expression during the in vitro studies, in an attempt to identify new virulence factors that could present a biological application against leishmaniasis. For this, the in vivo validation of this selected candidate was performed in a murine model against *L. infantum* infection. Our results pointed to one protein, the small myristoylated protein-3 (SMP-3), which showed low structural homology to human proteins, as well as an immunogenic role in PBMCs collected from healthy subjects and VL patients, which were stimulated with this recombinant antigen. In addition, SMP-3 was also immunogenic in BALB/c mice, when associated with saponin, by inducing a specific Th1 response that was responsible for protection when a challenge infection was performed using *L. infantum* promastigotes. As a consequence, a possible application of this molecule as a future vaccine against human disease can be considered.

## 2. Results

### 2.1. Computational Strategy to Predict Virulence Factors in L. amazonensis Proteins

A computational screening approach was designed to identify *Leishmania* virulence factors and test them as vaccine candidates against leishmaniasis in mammalian hosts [17]. The amino acid sequences of the 56 proteins were submitted to a functional annotation protocol. For this, the PSORTb, PSLpred, CELLO, WolfPsort, SignalP 4.1, SecretomeP, TMHMM, SUPERFAMILY, CATH, SMART, and InterPRO servers, which were combined with a statistical estimation of diagnostic accuracy, were used to produce a functional annotation. Among these molecules, seven were classified as hypothetical in the previous study. It was possible to successfully determine that the function and subcellular localization of four of these hypothetical proteins presented high average accuracy, sensitivity, and specificity values of 78.5%, 78.5%, and 100%, respectively (Table 1). Their sequences were functionally annotated as (WD40 repeat-like protein (Uniprot ID: E9ASM0)), (Phosphoenol pyruvate-like protein (Uniprot ID: E9AXT3)), (AlbA-like protein (Uniprot ID: E9B549)), and (Leucine rich-like protein (Uniprot ID: E9ATK7)), and were predicted to be parasite cytoplasmic proteins.

**Figure 1:**
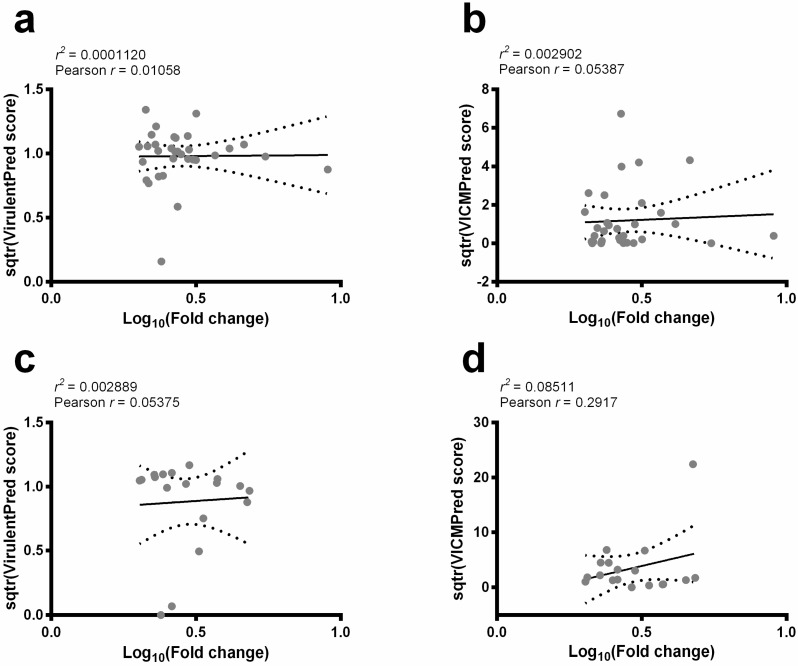
Linear regression plots and Pearson’s correlation coefficient. The Virulentpred and VICMpred servers were used to predict *Leishmania* virulence factors among the evaluated sequences. Their scores were transformed (*sqtr function*) and plotted against fold change in protein expression (*Log*_10_). The linear regression assays resulting in the proteins that decrease (**a**,**b**) or increase (**c**,**d**) their expression content are shown here. Solid line: linear regression; dotted lines: 95% confidence intervals.

The VICMpred and Virulentpred servers were used to predict virulence factors among the 56 selected sequences. The correlation between the fold change in their expression content and scores obtained from servers were also analyzed. There was no significant correlation between the decrease (Figure 1a,b) or increase (Figure 1c,d) in fold change in protein expression and the calculated scores by both servers. Nevertheless, consensual positive results identified three protein sequences that decreased as possible *Leishmania* virulence factors, and their sequences were selected for further analysis. In fact, screening the vaccine candidates for sequence homology with the human sequences allows for the identification and removal of those antigens that may cause a risk of inducing autoimmunity [18]. In this context, the structural homology to human proteins was evaluated in these candidates. The HHpred analysis showed that SMP-3 presented no significant homology to these proteins, and it was then selected to be validated as a potential vaccine candidate against disease (Table 2). A BLAST analysis was performed and results showed that *L. amazonensis* SMP-3 presents 91%, 98%, and 99% of structural homology among *L. braziliensis, L. major*, and *L. infantum* species, respectively.

### 2.2. Bioinformatics Assays and Cytokine Response Using Human PBMCs

**Figure 2:**
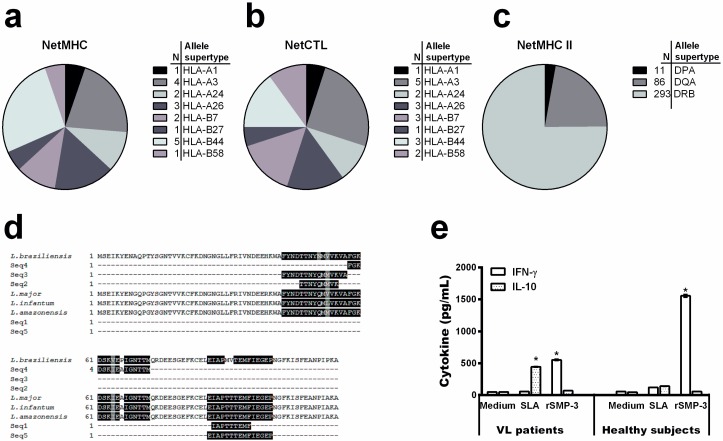
Mapping MHC class I and class II-specific T-cell epitopes. The chart shows the number of predicted epitopes through the NETMHC (**a**), NETCTL (**b**), and NETMHC Class II (**c**) servers for each allele supertype. The protein sequence in the *L. amazonensis, L. infantum, L. major,* and *L. braziliensis* species were then aligned, using the predicted T-cell epitopes, by the Clustal Omega program (**d**). The identical residues (black color), and the conservative (gray color) and semi-conservative (white color) substitutions are shown above. The cytokine response in PBMCs purified from healthy subjects living in an endemic area of leishmaniasis and VL patients was evaluated (**e**). Cells were non-stimulated (medium) or stimulated with rSMP-3 or *L. infantum* SLA (5.0 and 25.0 µg/mL, respectively), for 48 h at 37 °C in 5% CO_2_. IFN-γ and IL-10 levels were measured in the cell supernatant by an ELISA capture. Bars represent mean ± standard deviation. * indicates statistically significant differences (*p* < 0.005) between IFN-γ and IL-10 levels.

To evaluate the SMP-3 sequence regarding the presence of T-cell epitopes, the NETMHC and NETCTL servers were used to predict MHC I-specific epitopes, while the NETMHC class II server was applied to identify MHC II-specific epitopes. A total of 19, 20, and 390 epitopes were found by using the NETMHC (Figure 2a), NETCTL (Figure 2b), and NETMHCII (Figure 2c) programs, respectively. Since the total epitope prediction includes some degree of redundancy, a clustering step was performed. This analysis resulted in two and three sequences for MHC class I and II-specific epitopes. In addition, a sequence alignment was performed to select the best epitopes that belong to conserved regions of SMP-3, and results showed that IAPTTTEMF and TTNYQMMVK epitopes for MHC class I and FGKDSKIEAIGNTTM and EIAPTTTEMFIEGEP epitopes for MHC class II were those presenting higher coverage degrees between *L. infantum*, *L. major*, and *L. braziliensis* species (Figure 2d).

In an attempt to evaluate the immunogenicity of rSMP-3 in human cells, PBMCs collected from healthy subjects living in an endemic area of disease and VL patients were non-stimulated or separately stimulated with rSMP-3 or a soluble *Leishmania* antigenic extract (SLA). After, the cell supernatant was collected and used to evaluate the protein and parasite-specific IFN-γ and IL-10 production. In the results, high IFN-γ production was found when the recombinant protein was used to stimulate cells from both subjects, although higher production was found when PBMCs collected from healthy subjects were used (Figure 2e). On the other hand, a low IL-10 production was obtained when rSMP-3 was employed as a stimulus of cells from both individuals. Using SLA as a stimulus, a low IFN-γ and IL-10 production was found when PBMCs collected from healthy subjects were used, although IL-10 levels were higher when cells from VL patients were employed in the experiments.

### 2.3. In Vivo Validation against L. infantum Infection 

**Figure 3:**
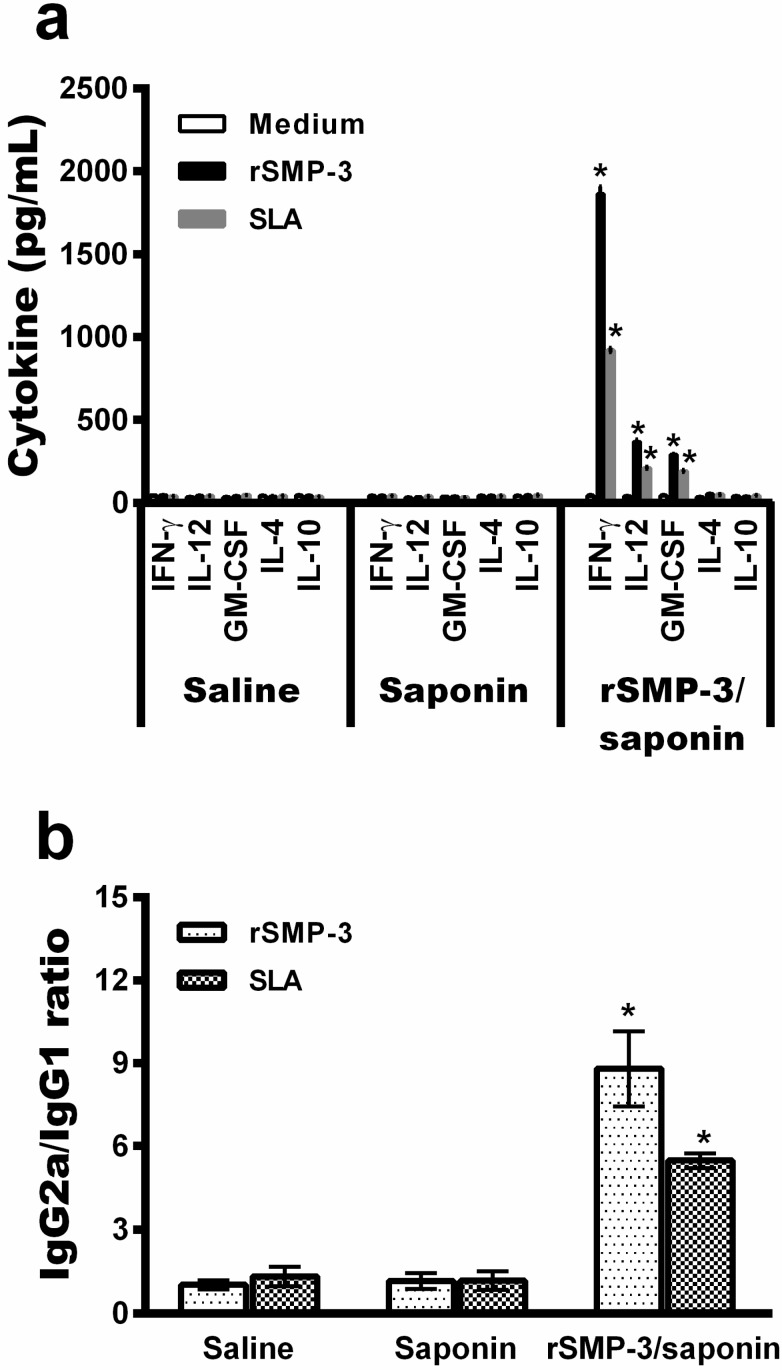
Cellular and humoral response induced in BALB/c mice before infection. Spleen cells were obtained from experimental mice 30 days after the last vaccine dose and before infection. These were cultured in the absence (medium) or presence of rSMP-3 or SLA (5 and 20 µg/mL, respectively). IFN-γ, IL-12, GM-CSF, IL-4, and IL-10 levels were measured by capture ELISA in the cell supernatants (**a**). The SMP-3 and parasite-specific IgG1 and IgG2a isotype levels were determined, and ratios between IgG2a and IgG1 were calculated and are shown (**b**). Bars represent the mean ± standard deviation. * indicates statistically significant differences (*p* < 0.005) between the rSMP-3/saponin group and the control (saline and saponin) groups.

To validate our computational screening, in which SMP-3 was identified as a possible *Leishmania* virulence factor, this protein was cloned and its recombinant version, together with saponin as an adjuvant, was administered in BALB/c mice. Animals followed a vaccination schedule of 30 days after the last vaccine dose and before challenge, as well as 60 days after *L. infantum* infection. After, the animals were euthanized to collect the blood and organs, aimed at evaluating parasitological and immunological parameters. The rSMP-3/saponin-vaccinated mice produced high levels of protein and parasite-specific IFN-γ, IL-12, and GM-CSF before infection (Figure 3a), which was associated with a higher production of the IgG2a isotype than the IgG1 molecule (Figure 3b), thus showing a Th1 immune response in these animals.

**Figure 4:**
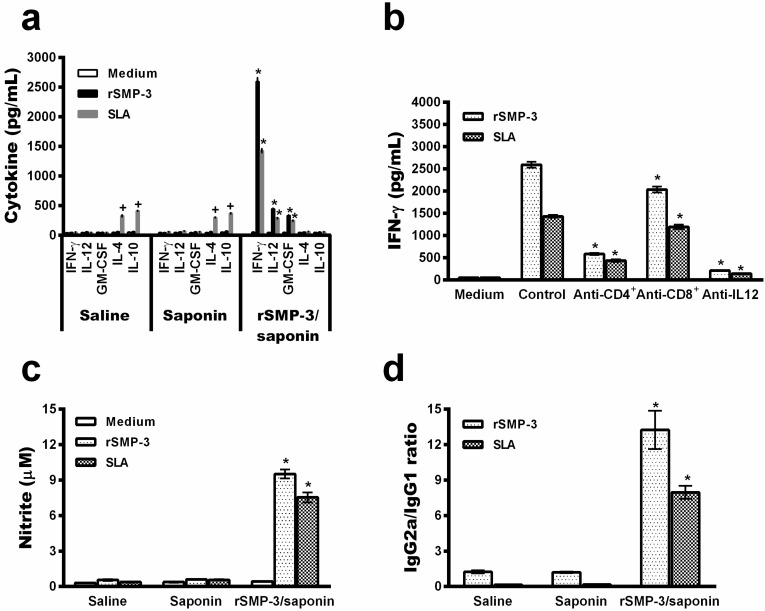
Analysis of the cell and humoral response induced by the rSMP-3/saponin vaccine in mice after challenge infection. Spleen cells were non-stimulated (medium) or stimulated with rSMP-3 or *L. infantum* SLA (5.0 and 20.0 µg/mL, respectively) for 48 h at 37 °C in 5% CO_2_. IFN-γ, IL-12, IL-4, and IL-10 levels were measured in cell supernatants by ELISA capture (**a**). The analysis of the involvement of IL-12 and CD4^+^ and CD8^+^ T cells in the IFN-γ production is shown (**b**). Levels of this cytokine found in the cell supernatants stimulated with rSMP-3 or SLA, as explained above, in the absence (positive control) or presence of anti-IL-12, anti-CD4, or anti-CD8 monoclonal antibodies were measured. The nitrite production was also evaluated by the Griess reaction (**c**). In addition, the humoral response in that SMP-3 and parasite-specific IgG1 and IgG2a isotypes levels were determined, and ratios between IgG2a/IgG1 levels are shown (**d**). In all cases, bars represent mean ± standard deviation. * indicates statistically significant differences (*p* < 0.005) between the rSMP-3/saponin group and the control (saline and saponin) groups. + indicates statistically significant differences (*p* < 0.005) between the control (saline and saponin) groups and the group rSMP-3/saponin.

After challenge infection, the immune profile was maintained in the vaccinated group mice, since higher levels of protein and parasite-specific IFN-γ IL-12, and GM-CSF were found, while in the saline and saponin groups, animals produced higher levels of IL-4 and IL-10, which proved to be compatible with the development of the Th2 response (Figure 4a). IFN-γ production in the infected and rSMP-3/saponin-vaccinated mice proved to be due mainly to the action of CD4^+^ T cells, since the use of a monoclonal antibody inhibition by neutralizing these cells was associated with lower levels of this cytokine in the cell supernatant (Figure 4b). The rSMP-3/saponin-vaccinated mice showed a high nitrite production, possibly indicating that this molecule could be involved in macrophage activation, consequently explaining, at least in part, the protection against *Leishmania* infection observed in these animals (Figure 4c). Corroborating the cell response, the higher production of IgG2a antibodies was maintained in the infected and vaccinated group. By contrast, a higher production of parasite-specific IgG1 was observed in the controls (Figure 4d).

**Figure 5:**
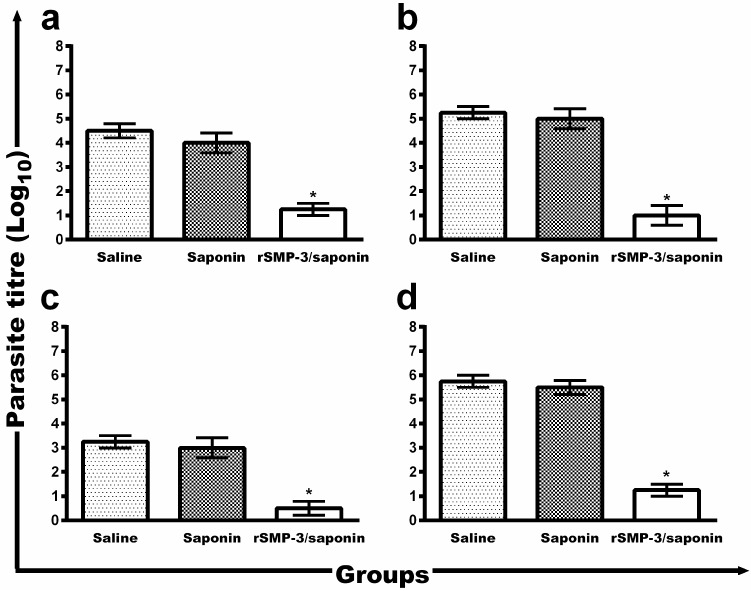
Parasite load in the infected and immunized BALB/c mice. The parasite burden in the animals (*n* = 8, in each group) was evaluated in the liver (**a**), spleen (**b**), bone marrow (**c**), and paws’ draining lymph nodes (**d**) by the limiting-dilution technique. Bars represent mean ± standard deviation. * indicates statistically significant differences (*p* < 0.005) between the rSMP-3/saponin group and the control (saline and saponin) groups.

To evaluate the protective efficacy induced by rSMP-3/saponin immunization, the parasite load was determined (Figure 5). The vaccine was effective in protecting against infection, with significant reduction in the parasite load in infected and rSMP-3/saponin-vaccinated mice, when compared to the mice from the saline and saponin groups: liver (3.3- and 2.8-log reductions, respectively), spleen (4.3- and 4.0-log reductions, respectively), draining lymph nodes (dLNs) (4.5- and 4.3-log reductions, respectively), and bone marrow (BM) (2.8- and 2.5-log reductions, respectively).

## 3. Discussion

*L. amazonensis* promastigotes were previously in vitro cultured for 150 days and the protein expression content of the parasites was evaluated by proteomics at different periods of time. Fifty-six proteins showed an increase or a decrease in their expression content within this time frame [17]. Some of these molecules that decreased their expression content have already been characterized as presenting an infective role in *Leishmania*, such as peroxidoxin [19], enolase [20], calreticulin [21], and tryparedoxin peroxidase [22]. Others showing an increase in their expression content were considered to be related to *Leishmania* metabolism [17]. However, the main issue is to determine whether these proteins are related to the infectivity of the parasites. In this context, the current study used a computational strategy to predict new virulence factors among these 56 previously identified proteins, as well as to validate their application, both in vitro and in vivo, as a vaccine candidate against leishmaniasis.

The maintenance of the *Leishmania* virulence is related to the in vivo infection sustained by parasite replication in mammalian hosts [17]. Others have also shown that in vitro cultivation for long periods leads to the loss of virulence in *L. infantum* [23] and *L. major* [24]. In the present study, proteins were evaluated by two virulence factor prediction servers, and the resulting scores were correlated with the fold change in their expression; however, no correlation was found. These results are in accordance with others, since studies have shown that the loss of virulence caused by the in vitro culture of promastigotes may well be the result of a growing inability to differentiate into amastigotes. Moreover, the induction of differentiation from promastigote to amastigote and then back to promastigote forms, both in vitro and in vivo, was capable of restoring *Leishmania* virulence [25]. Here, three sequences were predicted to be possible virulence factors and were submitted to a structural homology analysis against human proteins. Results showed that only SMP-3 presented low structural homology and was therefore selected for further immunological analysis.

Virulence factors in *Leishmania* have been proposed to involve two distinct groups of molecules: one consisting of invasive/evasive proteins and another comprised of conserved intracellular molecules, which can modulate interactions between parasite and host cells [26]. However, the relevance of these molecules in clinical manifestations found in the mammalian hosts has not yet been elucidated [27]. One of the main problems in applying bioinformatics tools to predict virulence factors in *Leishmania* has been based on the low number of available programs to predict these candidates in the parasites, since most of them use servers developed to identify virulent proteins in bacterial pathogens [28,29,30]. In our study, the VICMpred [31] and Virulentpred [32] servers, which are also applied to identify virulence molecules in these pathogens, were used to predict virulence factors in Leishmania. However, in our strategy, only proteins presenting satisfactory results using both servers were later evaluated to avoid false-positive results in the trials. In addition, the in vivo validation of the selected molecule, SMP-3, against *Leishmania* infection in a mammalian model allows one to draw a conclusion about the efficacy of the bioinformatics strategy employed in this study.

Myristoylated proteins present a key role in cell signaling, as well as in protein-protein and protein-membrane interactions [33]. In Trypanosomatids, 0.75% protein content in *L. major*, 0.76% in *T. brucei*, and 0.54% in *T. cruzi* are predicted to be myristoylated proteins [34]. However, only a few of these molecules have been identified and/or validated by experimental evidence, such as ADP-ribosylation factor-like protein, LdARL1, LdARL3A, and HASPB [35]. Another well-studied myristoylated protein is the small myristoylated protein-1 (SMP-1), which is located in the *Leishmania* promastigote flagellum and is responsible for stabilizing the flagellar membrane, either directly by forming a protein scaffold on the cytoplasmic leaflet or by increasing the degree of saturation of membrane lipids [36]. Moreover, myristoylation has been pinpointed as a step in initiating immune cell signaling cascades. As a consequence, myristoylation of a macrophage protein, namely MARCKS, is required for its effective co-localization with PKC, and critical for the production of inflammatory cytokines, such as TNF-α, GM-CSF and IFN-γ. In addition, it has been shown that myristoylation directly regulates the biological activity of endothelial nitric oxide, by placing this molecule in the plasma membrane, which is required for an efficient diffusion of NO in the extracellular space [37]. In addition, protein myristoylation plays with a role in the cytotoxic function of activated T cells, by means of the activation of the apoptotic mechanism in targeted cells, through the extrinsic pathway [38].

*Leishmania* vaccine research is a study area which requires further investigation, particularly given that available epidemiological and historical data indicate that an effective vaccine is a possible goal [39]. The development of a vaccine to prevent VL has been a long-term task for many studies, and there is an urgent need to find new immunity markers so that vaccine candidates may be effectively tested in mammalian models [40]. However, a limited number of antigens has showed immunogenicity and effective protection against *Leishmania* infection, as well as in distinct mammalian hosts [41,42,43]. Currently, it is possible to scan entire genomes searching for candidates and to select them to be tested as vaccines [44], and with the advent of reverse vaccinology, a significant effort has been made to provide predictor programs of new molecules with distinct biotechnological applications [45,46]. However, the bottleneck in this workflow analysis is the validation of predictions for protozoan parasites [47,48,49].

As a consequence, we tried to evaluate our protein candidate in a human model, by using PBMCs collected from both healthy subjects and VL patients. We have observed that, in both conditions, rSMP-3 was immunogenic in the stimulated human cells, thus demonstrating its feasibility to be used to immunize humans and to protect them against *Leishmania* infection. The defective cell immunity occurs in human VL with the inability of patient PBMCs to proliferate and/or produce Th1 cytokines, in turn contributing to the development of the disease [50]. Conversely, when these cells are collected from healthy people living in endemic areas of VL and stimulated in vitro by parasite antigens, a significant IFN-γ production is found, partly explaining their protection against infection [15]. In our study, rSMP-3 induced a Th1 response in PBMCs collected from both healthy subjects and VL patients, thus showing its potential immunogenic role.

An important aspect for vaccine development against leishmaniasis has also been based on the pre-clinical model used in the initial screening of the candidates. Although infection in hamsters using infected sand flies is considered to be more appropriate when studying molecules to be applied as human vaccines, this infection model usually requires sophisticated laboratory conditions and trained staff, which are not widely available, hampering its use as a first step for testing vaccine efficacy against disease [51]. On the other hand, BALB/c mice infected with *L. donovani* or *L. infantum* promastigotes is one of the most widely studied mammalian models for VL, and is therefore naturally selected over other models for this purpose [52,53,54]. A recent study reported that the subcutaneous route of *L. infantum* inoculation in these animals, when compared to the challenge using an intravenous route, can induce a similar infection development and parasite load in different organs of the animals [55]. In this context, the subcutaneous route was selected in the present study to evaluate the efficacy of our immunogen against *L. infantum* infection. Other studies have also shown protection through vaccination using intradermal and/or subcutaneous routes, when compared to those receiving an intravenous challenge [56,57]. Nevertheless, additional studies are warranted in an attempt to extend the findings described here by rSMP-3/saponin immunization to other infection models and experimental conditions.

To validate, in vivo, the immunogenicity and protective efficacy of this recombinant molecule against *Leishmania*, we have immunized BALB/c mice and challenged them with *L. infantum*. Resistant mice strains control parasite infection through the development of a Th1 immune response, marked by the production of inflammatory cytokines, such as IFN-γ, in response to IL-12 [50,58]. IFN-γ activates the leishmanicidal mechanisms in the infected phagocytes through the synthesis of inducible nitric oxide synthase (iNOS), which leads to the production of toxic radicals to kill internalized amastigotes. On the other hand, susceptibility has been associated with the development of a Th2 response, which is based on the production of cytokines, such as IL-4 and IL-10, and which inhibit the Th1 response and allow the disease to develop [59,60,61].

In the present study, a Th1 immune profile, based on high levels of protein and parasite-specific IFN-γ, IL-12, and GM-CSF, was found in rSMP-3/saponin-vaccinated mice, and this immune response was maintained after challenge infection. These cytokines were related to a high nitrite production, thus corroborating the antileishmanial mechanism induced by immunization using SMP-3. In addition, a significant reduction in parasitism was observed in different evaluated organs of the infected and vaccinated animals, when compared to the controls. In the vaccinated animals, not all parasites were eliminated. It is possible that a low level of *Leishmania* may persist in cured mammalian hosts, such as mice, dogs, and humans, allowing them to maintain the immune system continuously stimulated, in turn leading to an immunological memory and protection against reinfection [62,63]. Together with the results of immunogenicity from in vitro experiments using PBMCs, we propose that rSMP-3 should be considered a promising antigen for the development of vaccines against VL. Further studies are warranted.

## 4. Materials and Methods

### 4.1. Sequence Retrieval, Subcellular Localization, and Functional Annotation

A total of 56 proteins were evaluated, where 37 decreased and 19 increased in their expression content during in vitro cultivation. In addition, among these candidates, seven were identified as hypothetical proteins [17]. To predict their subcellular localization and functional annotation, an experimental strategy was employed as described [64]. Briefly, FASTA sequences of the proteins were retrieved from the UniProt database [65], and PSORTb 3.0 [66], PSLpred [67], CELLO [68], and WolfPsort [69] servers were used to predict the subcellular localization of the molecules. SignalP 4.1 [70] was used to predict signal peptides in the protein sequence, whereas Secretome P [71] was used to identify the involvement in the non-classical secretory pathway. TMHMM [72] was used to predict a molecule as a membrane protein, whereas the protein functional domain was predicted by SUPERFAMILY [73], CATH [74], SMART [75], and ProtoNet 6.0 [76] servers. Receiver Operator Characteristics (ROC) curves were constructed to evaluate the subcellular localization and to predict biological functions. Results were shown as sensitivity (Se), specificity (Sp), accuracy (Ac), and area under the curve (AUC).

### 4.2. Virulence Factors Prediction

Screening to identify *Leishmania* virulence factors among the evaluated proteins was performed by VICMpred [31] and Virulentpred [32] servers. Both programs are support vector machine-based methods used to predict virulence targets, showing an accuracy of 70.8% and 81.8%, respectively. The methods use a 5-fold cross-validation technique to evaluate different prediction strategies [77]. For this, FASTA sequences were uploaded to both servers, and sequences were considered to be virulence factors when the algorithms obtained a positive consensual result.

### 4.3. Protein Sequence Comparison 

The structural conservation between *Leishmania* and *Homo sapien* proteins was performed using the HHpred program, which employs a pair wise comparison of hidden Markov model profiles (HMMs) for remote protein homology detection by searching different databases [78]. Structural conservation was mapped onto the sequence alignment by the available Expresso options [79], and the BLAST tool was used to search for identity among the deposited sequences from non-redundant proteins [80]. Sequence alignment was performed using the Clustal Omega program with default parameters [81].

### 4.4. Mapping Linear MHC Classes I and II T-Cell Epitopes

The prediction of linear T-cell epitopes was performed using the NetMHC 4.0 [82] and NetCTL 1.2 [83] servers for MHC class I-specific epitopes, while the Net MHCII server was used to predict MCH class II-specific epitopes [84]. For this, the FASTA sequence of the proteins was uploaded to the servers, and the peptides that interacted with the highest number of alleles were selected. Information about MHC alleles was retrieved from the dbMHC database [85]. Considering the sequence similarity among the predicted epitopes, the IEDB epitope cluster analysis tool with a 70% level of identity was employed [86]. Threshold values for positivity were selected by default parameters.

### 4.5. Blood Samples, PBMCs Culture and Cytokine Assay

This study was approved by Human Research Ethics Committee of the Federal University of Minas Gerais (UFMG), logged under protocol number CAAE–32343114.9.0000.5149 (09/02/2014). Peripheral blood samples (20 mL) were collected, using heparin, from healthy subjects (*n* = 10, 6 males and 4 females presenting ages ranging from 23 to 49 years) living in an endemic area of VL (Belo Horizonte, Minas Gerais, Brazil). These subjects presented no clinical signs of disease and showed negative serological results when using commercial test kits (Kalazar Detect™ Test, InBios^®^ International, Seattle, WA, USA). Moreover, blood samples were collected from VL patients (*n* = 10, with 7 males and 3 females presenting ages ranging from 26 to 53 years) before beginning treatment. These patients showed clinical signs of disease and were diagnosed by the polymerase chain reaction (PCR) technique, targeting *L. infantum* kDNA, as well as by positive serology. PBMCs were purified by density centrifugation through Ficoll-Hypaque (GE Healthcare Bio-Sciences AB, Uppsala, Sweden). For this, cells (10^7^) were cultured in RPMI 1640 medium, together with 20% fetal bovine serum (FBS), 2 mM l-glutamine, 200 U/mL penicillin, 100 µg/mL streptomycin, 50 µM 2-mercaptoethanol, 1 mM sodium pyruvate, and 1× non-essential amino acid. Next, the PBMCs were plated in 48-well flat-bottomed tissue culture plates (Costar, Cambridge, MA, USA), and incubated in medium (control) or stimulated with rSMP-3 or *L. infantum* SLA (5.0 and 25.0 µg/mL, respectively) for 5 days at 37 °C in 5% CO_2_. The supernatants were then collected, and the IFN-γ and IL-10 levels were measured by capture ELISA (Human IFN-γ and IL-10 ELISA Sets, BD Biosciences, Franklin Lakes, NJ, USA).

### 4.6. Parasites, Mice, and Preparation of the Recombinant Antigen

The current study was also approved by the Committee on the Ethical Handling of Research Animals of UFMG (protocol number 333/2015) (12/09/2015). *L. infantum* (MOM/BR/1970/BH46) was used. Parasites were grown at 24 °C in Schneider's medium (Sigma-Aldrich, St. Louis, MO, USA), which was added with 20% heat-inactivated fetal bovine serum (FBS, Sigma-Aldrich) and 20 mM l-glutamine, pH 7.4. BALB/c mice (female, 8 weeks age) were maintained under specific pathogen-free conditions. The cloning, expression, and purification of the rSMP-3 protein was performed as described elsewhere [52]. After purification, the recombinant protein was passed on a polymyxin-agarose column (Sigma-Aldrich) to remove the residual endotoxin content (less than 10 ng of lipopolysaccharide per 1 mg of protein, measured by the Quantitative Chromogenic Limulus Amebocyte Assay QCL-1000, Bio-Whittaker, Walkersville, MD, USA).

### 4.7. Vaccination, Infection, and Cell Response

For immunization, BALB/c mice (*n* = 16 per group) were inoculated subcutaneously in their left hind footpad with 10 µg of the rSMP-3 plus 10 µg saponin (*Quillaja saponaria* bark saponin, Sigma-Aldrich, USA). Two doses were administered at 2-week intervals. Other animals (*n* = 16 per group) received saline or were immunized with saponin (10 µg). Thirty days after the last immunization, mice (*n* = 8 per group) were euthanized for the analysis of the immune response elicited by vaccination. At the same time, the remaining animals were infected subcutaneously in their right hind footpad with 10^7^ *L. infantum* stationary promastigotes and were followed up for 60 days, at which time they were euthanized and their spleens were collected for splenocyte cultures. Briefly, spleen cells (5 × 10^6^ per mL) were plated in 24-well plates and incubated in DMEM plus 20% FBS and 20 mM l-glutamine, pH 7.4, in the absence (medium) or presence of the rSMP-3 or SLA (5.0 or 20.0 µg/mL), for 48 h at 37 °C in 5% CO_2_. IFN-γ, IL-4, IL-10, IL-12p70, and GM-CSF levels were measured in cell supernatants using commercial kits (BD OptEIA TM set mouse, Pharmingen^®^, San Diego, CA, USA), while nitrite production was investigated by the Griess method. The participation of the CD4^+^ and CD8^+^ T cells in the IFN-γ secretion was evaluated in the rSMP-3/saponin group, using monoclonal antibodies against these molecules (IL-12 (C17.8), CD4 (GK 1.5), or CD8 (53–6.7), Pharmingen^®^), all in a concentration of 5 µg/mL, as described elsewhere [87,88].

### 4.8. Humoral Response

Antibody production was investigated before and after infection, during the same period of the splenocyte cultures. For this, rSMP-3 and *L. infantum* SLA (0.5 and 1.0 µg per well, respectively) were added to the plates for 16 h at 4 °C. After blocking with phosphate buffer saline (PBS 1×) plus 0.05% Tween 20 (PBS-T), individual serum samples were diluted 1:100 in PBS-T, and incubation was processed for 1 h at 37 °C. After washing, the anti-mouse IgG1 and IgG2a horseradish-peroxidase conjugated antibodies (Sigma-Aldrich) were diluted 1:5000 and 1:10,000, respectively, in PBS-T and added to the wells. A new incubation was processed for 1 h at 37 °C, at which time the plates were washed and reactions were developed using H_2_O_2_, ortho-phenylenediamine, and citrate-phosphate buffer pH 5.0, for 30 min, in the dark. Reaction interruption was performed using 2 N H_2_SO_4_, and optical density (O.D.) values were read in an ELISA microplate spectrophotometer (Molecular Devices, Spectra Max Plus, Sunnyvale, CA, USA), at 492 nanometers. The ratios between IgG2a and IgG1 levels were calculated as described elsewhere [87].

### 4.9. Evaluation of the Parasite Burden

Sixty days after infection, mice were euthanized and their liver, spleen, bone marrow (BM), and paws’ draining lymph nodes (dLNs) were collected for parasitological assays, which were performed using a limiting-dilution technique [80]. Briefly, organs were macerated and concentrated by centrifugation at 2000× *g*. The pellet was resuspended in 1 mL of Schneider’s medium together with 20% FBS. Next, 220 µL were plated onto 96-well flat-bottom microtiter plates (Nunc, Nunclon^®^) and diluted in log-fold serial dilutions in Schneider’s medium (10^−1^ to 10^−12^ dilution). Results were expressed as the negative log of the titer adjusted per milligram of organ, seven days after the beginning of the parasite culture at 24 °C. Experiments were repeated twice and presented similar results.

### 4.10. Statistical Analysis

Results were entered to Microsoft Excel (version 10.0, Microsoft Corporation, Redmond, WA, USA) spreadsheets and analyzed by GraphPad Prism^TM^ (version 6.0 for Windows, GraphPad Software, Inc., La Jolla, CA, USA). To evaluate the correlation between the fold change in the protein expression content and scores obtained from the virulence factor prediction, servers were transformed into a square root function (*sqtr*), while protein expression was transformed to a common logarithm (Log_10_). Subsequently, transformed data were placed in a linear regression plot and analyzed by Pearson’s correlation coefficient. Statistical analysis of the in vitro stimulation of PBMCs and the vaccination experiments was performed by using the one-way analysis of variance (ANOVA). Bonferroni’s post-test was applied for comparisons between the experimental groups. The mean ± standard deviation is shown, and differences were considered significant when *p* < 0.05.

## 5. Conclusions

This computational strategy was based on the application of bioinformatics tools to formulate a rational vaccine design to be tested in vivo against leishmaniasis. The results showed that a small myristoylated protein (SMP-3) was in silico predicted and validated as regards to its use as a vaccine candidate against *L. infantum* infection. Since this protein is conserved between different *Leishmania* species, it should be tested to protect against both tegumentary and visceral leishmaniasis in other mammalian models, such as dogs and humans, by using distinct immunization schedules, adjuvant systems, and/or administration routes, in an attempt to reduce the number of cases of this neglected disease worldwide.

## Figures and Tables

**Table 1:** List of functionally annotated *Leishmania* hypothetical proteins.

Uniprot ID	Gene ID	Predicted Function	Predicted Localization
E9ASM0	LMXM36.1000	WD40 repeat-like	Cytoplasmic
E9AXT3	LMXM25.2010	Phosphoenol pyruvate-like	Cytoplasmic
E9B549	LMXM33.2580	AlbA-like	Cytoplasmic
E9ATK7	LMXM36.4230	Leucine rich-like	Cytoplasmic

**Table 2:** List of structural homologies of the *Leishmania* protein sequences regarding human proteins.

Uniprot ID	Protein Name	Gene ID	Major HHpred Hit to *Homo sapien* Sequences		
			PDB	Probability (%)	*E*-Value
E9ATK7	WD40 repeat-like protein ^a^	LMXM36.4230	1Z7X	100	4.3 × 10^−29^
E9AQ29	Ribonucleoprotein p18	LMXM15.0275	4XGL	99.8	1 × 10^−17^
E9APT0	Putative small myristoylated protein-3	LMXM14.0850	2JX8	45.1	27

^a^ Non-characterized protein predicted as a WD40 repeat-like protein.
